# Cognitive impairment in Chinese adult patients with type III spinal muscular atrophy without disease-modifying treatment

**DOI:** 10.3389/fneur.2023.1226043

**Published:** 2023-11-03

**Authors:** Ying Hu, Ling Wei, Aonan Li, Tingting Liu, Yubao Jiang, Chengjuan Xie, Kai Wang

**Affiliations:** ^1^Department of Neurology, The First Affiliated Hospital of Anhui Medical University, Hefei, China; ^2^The School of Mental Health and Psychological Sciences, Anhui Medical University, Hefei, China; ^3^Anhui Province Key Laboratory of Cognition and Neuropsychiatric Disorders, Hefei, China

**Keywords:** neuromuscular diseases, spinal muscular atrophy, cognition, executive function, emotion

## Abstract

**Objective:**

Spinal muscular atrophy (SMA) is a neurodegenerative disorder characterized by the degeneration of motor neurons in the spinal cord. It remains uncertain whether the cognitive performance of adult patients with SMA is impaired. The objective of this study was to assess the cognitive profile of adult Chinese patients with SMA and the association between clinical features and cognitive ability, particularly executive function.

**Methods:**

This cross-sectional study included 22 untreated adult patients with type III SMA and 20 healthy subjects. The following variables were assessed: general intelligence, memory, attention, language, executive function, depression, anxiety, and other demographic and clinical parameters. In addition, physical function was evaluated using the Hammersmith Functional Motor Scale Expanded (HFMSE), the Revised Upper Limb Module (RULM), and the 6-Minute Walk Test (6MWT).

**Results:**

SMA patients had lower scores than healthy subjects in the Verbal Fluency Test, Stroop effect, Total Errors, Perseverative Responses, Perseverative Errors, and Non-perseverative Errors in the Wisconsin Card Sorting Test, showing impaired abilities of SMA patients in executive function. In the Attention Network Test (ANT), the results indicated that the SMA patients also had selective deficits in their executive control networks. Ambulant patients had better executive function test performance than non-ambulant ones. Compromised executive abilities in patients with SMA were correlated with a younger age at onset, poorer motor function, and higher levels of anxiety and depression.

**Conclusion:**

Our study presented the distribution of cognitive impairment in a Chinese cohort with SMA. Patients with type III SMA showed selective deficits in executive function, which may be associated with disease severity, physical impairment, depression and anxiety. Future cognitive studies, accounting for motor and emotional impairment, are needed to evaluate if executive impairment is driven by specific brain changes or by those confounding factors.

## 1. Introduction

Spinal muscular atrophy (SMA) is an autosomal recessive disease that affects motor neurons in the anterior horn cells of the spinal cord (1). SMA is considered one of the most frequent causes of infant morbidity and mortality, with the incidence being approximately 1 in 6000 to 10,000 newborns. In the Chinese population, the carrier frequency of SMA mutation is 1/42 (2). The most common form of SMA is caused by alterations in the survival motor neuron 1 (SMN1) gene, which is located at 5q11.2-q13.3. Traditionally, SMA is classified into 5 main subtypes (type 0, I, II, III, IV) based on the age of onset, severity of symptoms, and motor milestones (3). Specifically, type 0 is the most severe subtype with an onset during the prenatal period; type I is diagnosed within the first 6 months after birth, characterized by the inability to achieve independent sitting (4); type II usually has its onset between age 6 to 18 months, and patients with this type of SMA may be able to stand with support, but are not able to walk independently; type III is considered a juvenile form of SMA, and SMA type III patients can learn to walk independently, but may rely on a wheelchair during the course of their lives; type IV begins in adulthood (5).

In recent years, new treatment options, such as gene therapy that either modulates the splicing of SMN2 or replaces the mutated SMN1 gene, have dramatically changed the natural history of physical decline in patients with SMA (6). Most studies focused on the motor and respiratory outcomes of SMA patients, however, the cognitive development of SMA patients has not received much attention (7). It remains controversial whether patients with SMA have normal cognitive performance. Some of the previous studies supported the notion that SMA is solely a motor disease and therefore would not manifest as cognitive impairment. An early study even showed that the intelligence of children with SMA was generally in the normal range, and some of them were even above the average, suggesting a possible compensatory neuropsychological process (8). Researchers have speculated that such enhanced performance on a neuropsychological level may be attributed to a redistribution of cognitive resources, which might have otherwise been allocated to sensorimotor development (9). However, similar to Amyotrophic Lateral Sclerosis (ALS), which is characterized by a progressive deterioration and neurodegeneration of motor neurons, patients with SMA may also have cognitive deficits. Recent studies reported that the cognitive performance of SMA patients was below average (10, 11). Although SMA is widely known as a spinal motor neuron disease, recent evidence has indicated that it is a multiorgan disease with disease involvement of several non-motor regions (12).

Previous studies on the cognitive performance of SMA patients were largely conducted in Europe and North America, whereas no similar studies have been conducted in China, and the data for Chinese patients with SMA are rather limited (13, 14). Since 2001, multiple variants associated with SMA in Chinese patients have been identified, and the spectrum of subtle mutations in SMN1 among Chinese patients with SMA has been found to be distinct from that found in European and North American patients (15). The unique clinical characteristics and cultural backgrounds of Chinese patients may lead to potential differences in their cognitive profiles compared with European and North American patients. In addition, most of the previous studies focused on children and adolescents suffering from SMA; little research focused on the cognitive abilities in adult patients (16).

This cross-sectional study aimed to evaluate the cognitive function of adult patients with SMA and the association between clinical features and cognitive ability. We carried out a comprehensive assessment of the cognitive profile of Chinese adult patients with untreated SMA type III.

## 2. Materials and methods

### 2.1. Participants

This was an observational, cross-sectional study conducted at the Department of Neurology of the First Affiliated Hospital of Anhui Medical University, Anhui, China. Patients were aged ≥18 years, had genetically confirmed mutations in the SMN1 gene, and a diagnosis of type III 5q-SMA. Inclusion criteria were understanding of Chinese and being able to perform cognitive function testing that was written in Chinese. All the enrolled SMA patients had not received treatment with the antisense oligonucleotide nusinersen or other gene-based or disease-modifying therapies. Exclusion criteria included upper limb involvement that prevented completion of cognitive tasks and any neurological disease or condition related to long-term physical incapacitation. All these patients had received nusinersen treatment after the cognitive assessment.

The control group included a total of 20 age- and sex-matched healthy volunteers with average achievements within regular schools and their communities. None of the participants had any current or previous serious medical or neurological condition.

Written informed consent was obtained from all the participants.

### 2.2. Assessment of motor function, cognitive performance, depression and anxiety

Assessment of motor function was conducted by trained evaluators using the Hammersmith Functional Motor Scale Expanded (HFMSE), the Revised Upper Limb Module (RULM) assessment, and the 6-Minute Walk Test (6MWT) (17–19).

A neuropsychological test battery was used to evaluate various aspects of cognitions, including general intelligence, memory, attention, language, executive function, depression, and anxiety in the patients and control participants. The Montreal Cognitive Assessment (MoCA; ranging from 0 to 30, with higher scores indicating better cognitive function) was applied to assess general cognitive function (20). The MoCA can differentiate participants with mild cognitive impairment from the normal population, and it has a higher sensitivity in detecting cognitive decline than the Mini-Mental State Examination (MMSE) test (21). Verbal and executive functions were assessed using the Verbal Fluency Test (VFT), in which participants were asked to report as many items as possible in each category (categories: animals, fruits and vegetables, and water) within 60 s (22). The category “water” contained both semantic and phonemic fluency. Participants were asked to generate as many distinct Chinese characters as possible that began with the specific initial syllable and category within 60 s. For an instance, if the initial syllable is “shui” (which means water) correct answers include words such as “shuihu” (which means water bottle) and “shuihua” (which means splash of water). The Stroop Color–Word Interference test (SCWIT) is a well-established and common neuropsychological test that measures multiple dimensions of executive control, including error monitoring, working memory, selective attention, and inhibitory control (23). The test stimuli included images of colored dots (Part A), words unrelated to color presented in colored font (Part B), and color names presented in font colors different from the word (Part C; e.g., the word “red” in green font). In the SCWIT, the participants were asked to read 3 pieces of paper written in different colors and the time was recorded. The interference value was defined as the response time for Part C minus Part A. A higher score indicates greater interference. The Digital Span forward is used to measure attention and working memory span. The Digital span backward also is used to measure executive function. In this test, the participants were asked to recall number sequences in ascending numerical order (forward) or reverse numerical order (backward). Scores were calculated based on the number of recalled digits. A Chinese version of the Auditory Verbal Learning Test (AVLT) was performed to examine learning, recall, and recognition memory. The AVLT is a 15-item word-list learning and memory test that includes 5 learning trials with auditory presentation of the 15 words and immediate recall on each trial (List A Trials 1–5), an interference trial with a different list of words (List B), a short-delay free recall trial for List A words immediately after List B (Trial 6), a long-delay free recall trial for List A words after 30 min of unrelated testing (Trial 7), and delayed recognition of List A words presented among an equal number of new distractor words (Trial 8). Each right response is one point, and the maximum score is 15 for each trial (24).

**Figure 1:**
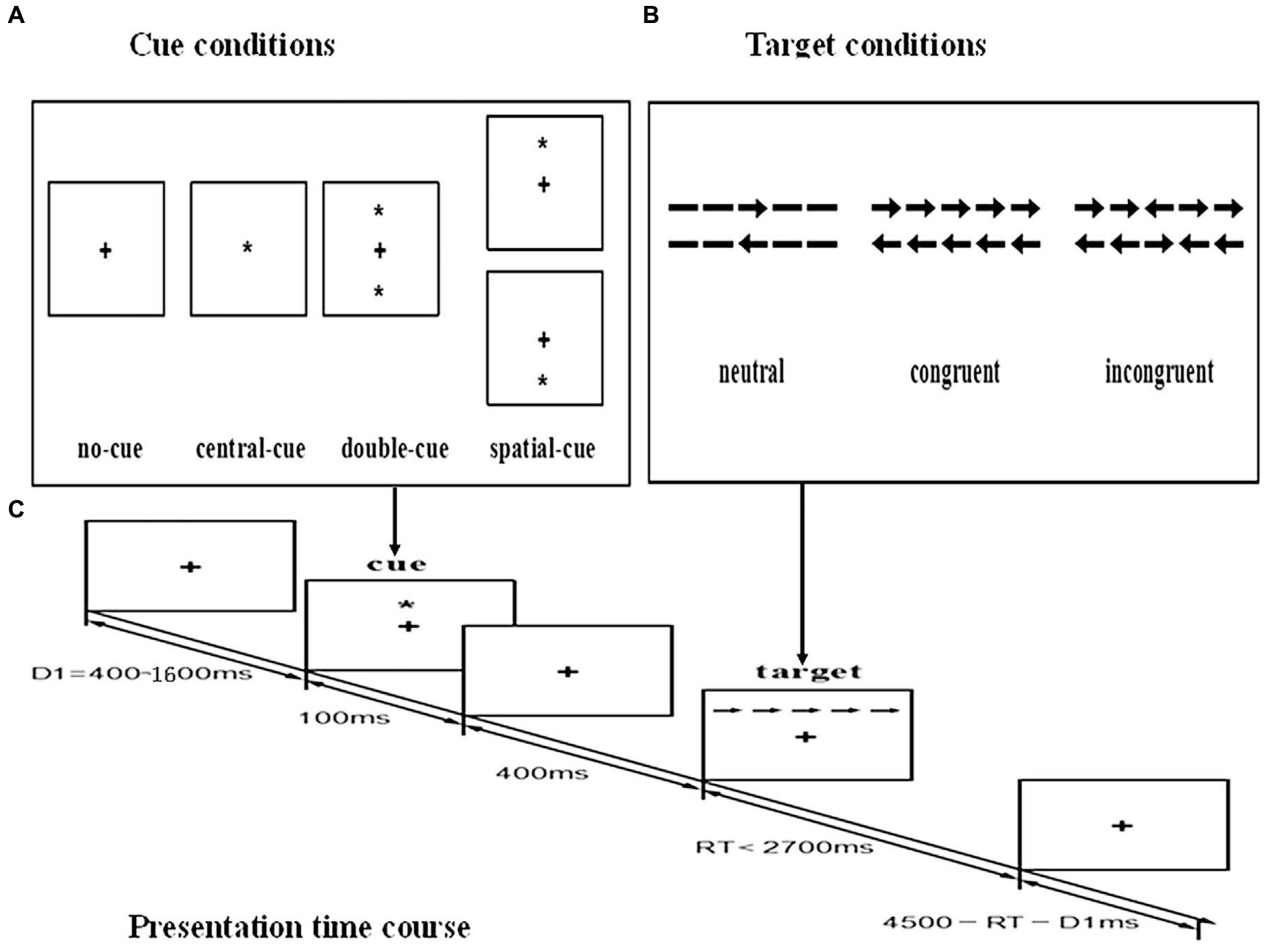
Schematic of the attention network test (ANT). **(A)** The four cue conditions. **(B)** The six stimuli used in the present experiment. **(C)** An example of the procedure.

The Attention Network Test (ANT) was used to measure attentional abilities. The human attention network model postulates 3 distinct functions of attention—alerting, orienting, and executive function subserved by three separate and relatively independent neural networks (25, 26). Since its creation in 2001, ANT has been widely used in over 300 studies to investigate attentional function both in healthy subjects and in patients with disorders such as schizophrenia, Alzheimer’s disease, and borderline personality disorder (27, 28). The details of ANT are shown in Figure 1. Participants viewed the stimuli shown on a computer screen, and responses were automatically collected via two response buttons. The stimuli consisted of a row of five horizontal black lines, with arrowheads pointing left or right, and the target was a left or right pointing arrowhead in the center, against a gray background. The target stimulus was flanked on either side by two arrows pointing either in the same direction (congruent condition), or in the opposite direction (incongruent condition), or by nothing (neutral condition). The participant’s task was to identify the direction of the center arrow. The target stimulus remained on the screen until the participant responded, but the maximum response time was cut off at 1700 ms. Cues consisted of an asterisk appearing for 100 ms, presented 400 ms before the presentation of the target. There were four cue conditions in the process: (1) no cue, the participant was shown across at the same location as the first stationary cross for 100 ms; (2) a center cue, an asterisk was presented on the central point; (3) a double cue, an asterisk was presented at two target locations simultaneously, above and below the central point; and (4) a spatial cue, an asterisk was presented at a target location either above or below the central point. Throughout the task, participants were required to concentrate on a centrally located fixation cross and respond by pressing the keyboard direction key as quickly and accurately as possible. The target item was flanked by either neutral, congruent, or incongruent arrows, and it was preceded by either a central-cue, double-cue, spatial-cue, or no-cue (see Figure 1). Efficiency of the alerting network was measured by changes in the reaction time (RT) for a warning cue, efficiency of the orienting network was measured by changes in the RT to cues indicating where the target will occur, and efficiency of the executive network was measured by asking subjects to indicate the direction of a central arrow surrounded by congruent, incongruent, or neutral flankers. For each participant, median RT and accuracy were computed.

In addition to the neuropsychological test battery, a computer version of the Wisconsin Card Sorting Test (WCST) was used to assess executive function. The test consists of four stimulus cards and 128 response cards that depict figures of varying forms, colors and numbers (29, 30). Before beginning the task, the experimenter indicates to the participant that the matching (sorting) rules cannot be explained in advance, and that they should try their best to make correct matches based on feedback (correct, incorrect) provided after each response. After a certain number of correct trials, the matching rule is changed without notice by the program. When the participant realizes this shift in the feedback has taken place, he/she has to determine the new sorting rule through trial and error. This process is repeated until categories have been successfully completed or until all of the cards had been administered. In the test, participants were required to sort cards that contained periodically changed stimuli and match the stimulus card with a key card according to an unspecified matching rule (31). The following indices were examined: categories completed, total errors, correct responses, number of trials, perseverative responses, perseverative errors, non-perseverative errors, and trials to complete the first category.

Depression and anxiety among patients with SMA were assessed using the Hamilton Depression Rating Scale (HAMD) and the Hamilton Anxiety Rating Scale (HAMA), with a HAMD score of >7 indicating depression and a HAMA score of >7 indicating anxiety (32, 33). Previous studies have found that HAMD and HAMA have good validity and reliability in Chinese population (34).

### 2.3. Statistical analysis

Statistical analyses were conducted using the IBM SPSS for Windows 17.0 package program. We examined all the outcome scores for normal distribution by means of Shapiro–Wilk tests. Descriptive statistics were shown in terms of absolute numbers and percentages for categorical data. Continuous variables were expressed as mean ± standard deviation (SD) for normally distributed data and median (25–75th percentile) for data that were not normally distributed. Equality of variances was tested by means of Levene’s test. When data failed to meet criteria for parametric analysis, nonparametric analysis was applied instead. Between-group differences in continuous variables were analyzed with either the Mann–Whitney test or the t-test, and differences in categorical variables were assessed using the chi-square test. Correlation analyses were performed using the Spearman’s correlation coefficient for metric variables.

## 3. Results

### 3.1. Demographic and clinical characteristics

A total of 22 patients (19 males and 3 females) with type III 5q-SMA and 20 healthy controls (17 males and 3 females) were enrolled between October 2021 and May 2023. The demographic and clinical characteristics are shown in Table 1. There were no significant differences between the 2 groups in age or years of education. All patients and controls had received at least 9 years of education and finished junior school in China. Of the 22 patients, 10 patients were using an electric wheelchair (sitters), while 12 patients were able to walk (walkers). They were all able to sit at the time of examination without the need for a sitting aid. The 12 walkers were in part-time employment and the remaining patients (sitters) were unemployed. Among the 20 healthy controls, 10 were employed and the remaining 10 were unemployed. All patients exhibited normal speech and swallowing abilities. None of them needed assisted ventilation or were fed by gastrostomy. No patient had received any approved or investigational SMN-restoring treatment for SMA.

**Table 1:** Demographic characteristics of SMA patients and healthy controls.

Characteristics	SMA III	Healthy controls	Test statistics	*p* values
Sample size, *n*	22	20		
Sex			*χ*^2^ = 0.016	*p* = 0.90
Female	3 (13.64%)	3 (15%)		
Male	19 (86.36%)	17 (85%)		
Age [years, median (IQR)]	30 (25–34)	27 (24–30)	*Z* = −1.724	*p* = 0.085
Education (years, mean ± SD)	13.45 ± 4.12	13.64 ± 2.91	*t* = −0.190	*p* = 0.850
Age at onset [years, median (IQR)]	11.5 (5–17)			
Disease duration [years, median (IQR)]	16 (14–25)			
SMN2 copy number, *n* (%)				
3	11 (50%)			
4	9 (40.9%)			
5	2 (9.1%)			
Current disease state, *n* (%)				
Walkers	12 (54.55%)			
Sitters	10 (45.45%)			
Motor function				
HFMSE score (mean ± SD)	38.75 ± 14.95			
RULM score [median (IQR)]	34.5 (26.75–37)			
6MWT score [*n* = 12, median (IQR)]	244 (158–425)			

SMA, spinal muscular atrophy; SMN2, survival motor neuron 2 gene; HFMSE, Hammersmith Functional Motor Scale Expanded; RULM, Revised Upper Limb Module; 6MWT, 6 min walking test.

### 3.2. Motor function

The motor function scores in patients with SMA are summarized in Table 1. Among the 12 walkers, the HFMSE score ranged from 22 to 58 points (median 44, interquartile range [IQR] 39–52) and the RULM score ranged from 33 to 37 points (median 37, IQR 35–37); the distance assessed in the 6MWT ranged from 131 to 427 m (median distance 244 m, IQR 158–425). In the 10 sitters, the HFMSE score ranged from 4 to 48 points (median 25, IQR 8–34) and the RULM score ranged from 5 to 34 points (median 26, IQR 17–29). The walkers showed significantly higher scores in the HFMSE (*U* = 11.0, *Z* = 2.985, *p* = 0.003) and RULM (*U* = 2.0, *Z* = −3,729, *p* < 0.001) than the sitters.

### 3.3. Neuropsychological cognitive tests

All the 22 patients with SMA and 20 controls completed the cognitive, behavior, anxiety, and depression assessments. The results of these tests are summarized in Table 2. There were no significant differences in scores for the MoCA, Digital Span, AVLT, and VFT for the category “animals” between the 2 groups. SMA patients performed worse than the healthy controls on the Stroop effect of SCWIT and VFT for the categories “fruits and vegetables” and “water” (*p* < 0.05). Based on the WCST, patients with SMA performed worse than healthy controls on Total Errors, Perseverative Responses, Perseverative Errors, and Non-perseverative Errors (*p* < 0.05). The difference in the Categories Completed between the 2 groups was marginally significant (*p* = 0.056). There were no significant differences between the 2 groups in the scores for other WCST subitems. Depression and anxiety scores in the HAMD and HAMA were higher in SMA patients compared with the healthy controls (*p* < 0.05).

**Table 2:** Values of neuropsychological test performances in SMA patients and healthy controls.

	SMA III	Healthy controls	Test statistics	*p* values
MoCA score [median (IQR)]	26 (26–27)	27 (26–27)	*Z* = −1.483	*p* = 0.138
AVLT immediate [median (IQR)]	9 (8.2–11.5)	11.2 (7.8–12.4)	*Z* = −0.535	*p* = 0.593
AVLT delayed [median (IQR)]	11 (9–12)	12 (11–14)	*Z* = −1.749	*p* = 0.080
AVLT recognition [median (IQR)]	14 (14–15)	14.5 (14–15)	*Z* = −1.207	*p* = 0.228
DS forward [median (IQR)]	9 (7–10)	8.5 (8–9)	*Z* = −0.080	*p* = 0.936
DS backward [median (IQR)]	6 (5–8)	7 (6–8)	*Z* = −1.273	*p* = 0.203
VFT animals [median (IQR)]	23 (21–24)	23 (21–25)	*Z* = −0.479	*p* = 0.632
VFT fruits and vegetables [median (IQR)]	17 (15–18)	19 (17–23)	*Z* = −2.131	*p* = 0.033^*^
VFT water [median (IQR)]	5 (4–7)	9 (6–10)	*Z* = −2.911	*p* = 0.004^**^
Stroop interference [median (IQR)]	17.0 (12.6–20.0)	10.5 (6.7–12.5)	*Z* = −4.041	*p* < 0.001^**^
HAMD (mean ± SD)	7.2 ± 3.71	3.2 ± 1.9	*t* = 4.664	*p* < 0.001^**^
HAMA (mean ± SD)	8.1 ± 4.5	3.2 ± 2.1	*t* = 4.506	*p* < 0.001^**^
WCST				
Categories Completed [median (IQR)]	8 (8–9)	9 (8–9)	*Z* = −1.909	*p* = 0.056
Total Errors [median (IQR)]	24 (19–29)	19 (15–25)	*Z* = −2.067	*p* = 0.039^*^
Correct Responses [median (IQR)]	99 (96–103)	100(96–103)	*Z* = −0.737	*p* = 0.461
Number of Trials [median (IQR)]	128 (118–128)	121 (116–128)	*Z* = −1.631	*p* = 0.103
Perseverative Responses (mean ± SD)	41.4 ± 6.6	36.2 ± 4.9	*t* = 3.178	*p* = 0.003^**^
Perseverative Errors [median (IQR)]	14 (11–17)	12 (10–13)	*Z* = −2.173	*p* = 0.030^*^
Non-perseverative Errors (mean ± SD)	9.2 ± 2.9	6.9 ± 2.8	*t* = 2.806	*p* = 0.007^**^
Trials to 1st category [median (IQR)]	14 (11–19)	12 (11–16)	*Z* = −0.254	*p* = 0.799

SMA, spinal muscular atrophy; MoCA, the Montreal Cognitive Assessment; AVLT, Auditory Verbal Learning Task; DS, Digital Span; VFT, Verbal Fluency Test; HAMD, Hamilton Depression Scale; HAMA, Hamilton Anxiety Scale; WCST, Wisconsin Card Sorting Task. Significance levels: ^*^p < 0.05; ^**^p < 0.01.

### 3.4. Performance on the attention network test

The scores for each of the attention networks, RT, and global accuracy are summarized in Table 3. The mean RT for the executive control network of the SMA group was significantly longer than that of the healthy controls (*p* < 0.05). There was no significant difference in terms of the RT for the alerting component and the orienting component between the 2 groups. Additionally, no significant differences in the overall mean RTs and global accuracy of the SMA group and healthy controls were observed.

**Table 3:** Attention network scores of the SMA patients and healthy controls.

	SMA III	healthy controls	Test statistics	*p* values
Alerting [ms, (mean ± SD)]	32.1 ± 21.7	40.7 ± 20.3	*t* = −1.431	*p* = 0.159
Orienting [ms, (mean ± SD)]	48.6 ± 16.0	45.8 ± 20.4	*t* = 0.536	*p* = 0.594
Executive [ms, median (IQR)]	117 (108–132)	104 (88–115)	*Z* = −2.788,	*p* = 0.005^**^
Mean RT [ms, (mean ± SD)]	617.9 ± 61.8	613.0 ± 68.2	*t* = 0.263	*p* = 0.793
Accuracy [%, median (IQR)]	99 (98–99)	99(98–99)	*Z* = −0.894	*p* = 0.371

ms, milliseconds; RT, reaction time. Significance levels: ^**^p < 0.01.

### 3.5. Comparison between walkers and sitters

We compared baseline characteristics and test scores between walkers (*n* = 12) and sitters (*n* = 10) among the SMA patients. The results of the tests are summarized in Table 4. The walkers showed significantly higher age (*U* = 12.0, *Z* = −3.203, *p* = 0.001), age at onset (*U* = 4.0, *Z* = −3.711, *p* < 0.001), and education (*U* = 28.0, *Z* = −2.147, *p* < 0.001) than the sitters. The walkers also performed better in some WCST subitems, for example, the Trials to 1st category (*U* = 14.0, *Z* = −2.883, *p* = 0.004) and Non-perseverative Errors (*U* = 15.0, *Z* = −2.797, *p* = 0.005), showing a better executive function than the sitters. No significant differences between the 2 groups were found in other cognitive domains, including the MoCA, AVLT, Stroop effect, Digital Span, VFT, ANT, HAMD, and HAMA.

**Table 4:** Values of neuropsychological test performances in SMA walkers and sitters.

	SMA Walkers (*n* = 12)	SMA Sitters (*n* = 10)	Test statistics	*p* values
Age [years, median (IQR)]	31 (30–35)	27 (18–30)	*Z* = −3.203,	*p =* 0.001^**^
Age at onset [years, median (IQR)]	16 (12–18)	5 (2–6)	*Z* = −3.711	*p <* 0.001^**^
Disease duration [years, median (IQR)]	16 (14–23)	23 (12–27)	*Z* = −0.683	*p =* 0.495
Education [years, median (IQR)]	16 (12–19)	9 (9–16)	*Z* = −2.147	*p* < 0.001^**^
MoCA score [median (IQR)]	26 (26–28)	26 (25–27)	*Z* = −1.296	*p* = 0.195
AVLT immediate [median (IQR)]	8.6 (8.2–11.6)	10.6 (9–11.2)	*Z* = −0.743	*p* = 0.458
AVLT delayed [median (IQR)]	11 (9–12)	11 (11–12)	*Z* = −0.126	*p* = 0.900
AVLT recognition [median (IQR)]	14 (12–15)	14 (14–14)	*Z* = −0.264	*p* = 0.792
DS forward [median (IQR)]	9 (7–10)	9 (7–10)	*Z* = −0.190	*p* = 0.850
DS backward [median (IQR)]	7 (5–8)	6 (6–7)	*Z* = −0.25	*p* = 0.802
VFT animals [median (IQR)]	23 (21–25)	22 (21–24)	*Z* = −1.55	*p* = 0.119
VFT fruits and vegetables [median (IQR)]	18 (16–20)	17 (15–18)	*Z* = −0.997	*p* = 0.319
VFT water [median (IQR)]	6 (5–8)	5 (4–7)	*Z* = −0.870	*p* = 0.384
Stroop interference [median (IQR)]	17 (12–20)	16.9 (9–27)	*Z* = −0.185	*p* = 0.853
HAMD [media (IQR)]	7 (2–8)	9 (5–13)	*Z* = −1.553	*p* = 0.121
HAMA [media (IQR)]	9 (4–12)	9 (4–12)	*Z* = −0.186	*p* = 0.852
WCST				
Categories Completed [median (IQR)]	9 (8–9)	8 (8–9)	*Z* = −0.922	*p* = 0.356
Total Errors [median (IQR)]	23 (19–27)	29 (19–35)	*Z* = −1.661	*p* = 0.097
Correct Responses [median (IQR)]	100 (96–104)	98 (93–102)	*Z* = −1.263	*p* = 0.207
Number of Trials [median (IQR)]	128 (113–128)	128 (120–128)	*Z* = −0.639	*p* = 0.523
Perseverative Responses [media (IQR)]	41 (40–46)	40 (34–48)	*Z* = −0.266	*p* = 0.791
Perseverative Errors [median (IQR)]	15 (11–16)	16 (12–23)	*Z* = −0.928	*p* = 0.354
Non-perseverative Errors [media (IQR)]	8 (7–9)	12 (9–13)	*Z* = −2.797	*p* = 0.005^**^
Trials to 1st category [median (IQR)]	12 (10–14)	20 (14–22)	*Z* = −2.883	*p* = 0.004^**^
ANT				
Alerting [ms, median (IQR)]	33 (31–52)	20.5 (0–52.3)	*Z* = −1.590	*p* = 0.112
Orienting [ms, median (IQR)]	49 (33–55)	44 (31–61)	*Z* = −0.398	*p* = 0.690
Executive [ms, median (IQR)]	113 (108–119)	122 (114–143)	*Z* = −1.393	*p* = 0.164
Mean RT [ms, median (IQR)]	580 (558–612)	656 (600–685)	*Z* = −1.587	*p* = 0.113
Accuracy [%, median (IQR)]	99 (99–99)	99 (98–99)	*Z* = −1.619	*p =* 0.105

SMA, spinal muscular atrophy; MoCA, the Montreal Cognitive Assessment; AVLT, Auditory Verbal Learning Task; DS, Digital Span; VFT, Verbal Fluency Test; HAMD, Hamilton Depression Scale; HAMA, Hamilton Anxiety Scale; WCST, Wisconsin Card Sorting Task; ms, milliseconds; RT, reaction time. Significance levels: ^*^p < 0.05; ^**^p < 0.01.

### 3.6. Correlation analyses

Correlation analyses indicated that the Stroop scores were significantly correlated with the HAMD (rho = 0.475, *p* = 0.019) and HAMA (rho = 0.560, *p* = 0.004) scores among the SMA patients. The details are displayed in Supplementary Table 1. Significant correlations were also observed between ANT executive control scores and the HAMD scores (rho = 0.692, *p* < 0.001), and the HAMA scores (rho = 0.553, *p* = 0.005) among the SMA patients. On the other hand, the age at onset was significantly correlated with the Trials to 1st category (rho = −0.538, *p* = 0.007), and the Non-perseverative Errors in the WCST (rho = −0.441, *p* = 0.031). Years of education were also significantly correlated with the Trials to 1st category (rho = −0.448, *p* = 0.022), and the Non-perseverative Errors (rho = −0.391, *p* = 0.048) in the WCST. The RULM scores were significantly correlated with the Trials to 1st category (rho = −0.586, *p* = 0.003) in the WCST. The 6MWT scores were also significantly correlated with the correct responses (rho = 0.607, *p* = 0.021) in the WCST. However, no correlations between these clinical variables and cognitive scales were found among the healthy controls.

## 4. Discussion

The present study added to the limited body of evidence on the cognitive function and psychological state of adult patients with type III SMA. To the best of our knowledge, this was the first study investigating potential cognitive alternations in Chinese adults with SMA. In particular, none of these patients had received any disease-modifying treatments, which excludes the treatment effect. Data on the cognition performance for this group were scarce before this study. Overall, our findings demonstrated that the SMA patients performed worse in some categories of VFT, Stroop effect, Total Errors, Perseverative Responses, Perseverative Errors, and Non-perseverative Errors in the WCST, showing impaired abilities in executive function; however, patients with SMA showed no significant differences in MoCA, AVLT, Digital Span, and other subitems of WCST compared to the healthy control group. The ANT test also indicated that the SMA patients had selective deficits in the executive control network. These results showed that the SMA patients spent more time resolving conflicts compared to the healthy control group. We also found that for patients with type III SMA, the walkers had better executive function than the sitters. Additionally, correlation analysis showed that the decline in executive function was related to patients’ age at onset, motor function, anxiety, and depression.

Previous studies reported inconsistent results on the cognitive performance among children and adolescents with SMA types I–III, which should be interpreted in view of highly heterogeneous samples in terms of age and SMA type (14). In a large study on intelligence and cognitive functions in 96 children and adolescents with chronic type I–III SMA, patients had a general intelligence within the normal range (35). It was speculated that, in SMA patients, normal intelligence was achieved through advanced creativity and knowledge developed during childhood to compensate for impaired motor ability. However, with the development and application of new cognitive evaluation protocols, poor cognitive performance was frequently reported in recent studies. Studies where children with SMA showed cognitive performance below average demonstrated that there was a high risk of impaired attention and executive function in these patients (14). In a cohort of 83 SMA children in Hong Kong, their parents were interviewed to score the child’s functional independence measure in daily activities, which also required executive function (36). In this assessment, a significant proportion of patients still needed assistance or supervision in their social interaction and problem solving.

Existing data on the cognitive performance of adult patients with SMA remained limited. A recent study compared the cognitive performance of adult SMA patients with that of patients with amyotrophic lateral sclerosis (ALS) (37). Using the Edinburgh Cognitive and Behavioral ALS Screen (ECAS) and a German vocabulary test, the authors found that SMA patients performed better than ALS patients in the cognitive domains of memory, language, and executive function. However, in this study, a large number of SMA patients scored below the cut-off values, which suggested a possible impact of SMA on cognition, similar to the cognitive impairment in ALS. Of note, the ECAS might not be sensitive enough to detect subtle cognitive alterations in SMA patients (11). Interestingly, similar to ALS, SMA exhibits SMN protein deficiency on the pathogenetic molecular pathway that is thought to play a crucial role in brain development (38). In a study by Kizina et al. the Wechsler Adult Intelligence Scale (Fourth edition), an internationally recognized and validated intelligence quotient (IQ) test, was used to measure major intelligence components of adult SMA patients (10). In their study, SMA type III patients showed a trend of lower IQ scores. These results may refute the widespread hypothesis that SMA patients could improve their cognitive skills and knowledge to compensate their physical handicaps (39). Our study showed no significant differences in the MoCA test between the adult SMA patients and healthy control group. One possible explanation is that the simplified cognitive performance tests, MoCA or the MMSE, are mainly used as screening tools and are not suitable for identifying minor cognitive dysfunction. However, decreased cognitive performance was observed in more precise and subtle cognitive domains, especially in most of the popular executive function measures. Consistent with our findings, Lenzoni et al. reported poorer performance of SMA patients in terms of executive functions and language (11). In their SMA samples, greater motor difficulties were associated with worse performance in attention and working memory. Taken together, our results are in line with previous findings of impaired cognitive function in selective domains. As impaired motor function may affect the assessment results of executive function in patients with SMA, further research on cognitive function in these patients that adjusts for motor disability is required to validate these limited findings.

Executive function refers to a set of high-order cognitive abilities that depend on top-down control processes in the brain (40). The ANT, which provides assessment of all the 3 functions of attention in 1 relatively short task, has been widely used as a behavioral test to evaluate performance of patients with neuropsychological and neurodegenerative diseases (41). The test can easily be performed even by patients affected by movement disorders (42). However, no study has reported the ANT results in patients with SMA. In our research, the RT for the executive control component in the SMA group was significantly longer than that for the healthy control group. We speculated that the decreased attentional function of these patients might be due to their insufficient executive function. Studies have revealed that the prefrontal cortex plays a crucial role in supporting the executive control network and the deficit in the executive control component of the attention network is related to frontal lobe damage, such as brain tumors and lesions in this area (43). There is evidence indicating that the patients with prefrontal lesions have more errors and fewer categories achieved, higher levels of interference in Stroop tests, and lower scores in VFT (30, 44). Our results showed that SMA patients had more Total Errors, Perseverative Responses, Perseverative Errors, and Non-perseverative Errors in the WCST than the healthy control group. The SMA patients also showed a poor ability to vocalize a specific semantic category in the VFT and required a longer time to complete the Stroop tests. This indicates a possible relationship between the deficits in the executive function and potential brain changes in patients with SMA type III, which warrants further investigation.

SMA belongs to a neurodegenerative spectrum involving multiple systems. A lack of SMN protein in patients with SMA may not only affect spinal motor neurons but also other cellular compartments of the central nervous system (CNS). It was hypothesized that patients with a lower SMN2 copy number and less SMN proteins were more severely affected and consequently had a lower IQ (10). Immunohistochemistry revealed a widespread expression of SMN in the CNS in the normal population (45). Specific neuropathological abnormalities have been reported in several CNS areas other than lower motor neurons in patients with SMA. In an autopsy case of SMA type III, Kuru et al. verified that some of the cortical neurons appeared atrophic and eosinophilic (46). Another study that conducted pathological examinations of patients with SMA type II also revealed a reduced myelinated fiber density in the frontal white matter, pointing to the possibility of maldevelopment of the nervous system (47). A case study reported the results of computerized tomography scans of 8 children with SMA type I and showed generalized cerebral cortical atrophy (48). A recent multimodal magnetic resonance imaging study in adults with milder and slowly progressive SMA type III did not show cortical atrophy, but the researchers indicated compensatory grey matter changes (49). Patients with more severe clinical phenotypes present with more widespread features of neuronal degeneration, possibly occurring below a threshold of SMN protein (50). Although the precise clinical significance of these changes remains unknown, different pathological changes in the brain regions may influence patients’ cognitive performance, in particular, executive function, as shown in the present study. However, this hypothesis needs to be validated in future studies that include neuroimaging or neuropathology assessments. In addition, the physical and emotional impairments may also play important roles. It remains unclear whether cognitive impairment is primarily caused by neurodegeneration as a result of intrinsic brain changes or secondary to other confounding factors during the development of the disease.

The psychological impact for SMA patients is complicated by the progressive disease condition (51). Loss of mobility and loss of the use of limbs are determinants of physical work problems. They are being forced to deviate from the labor market. In patients with SMA, limited mobility and inability to socialize may lead to depression, and continuous attempts to adapt and adjust goals may cause enduring distress. In addition, they may live with anxiety about the future and progressive decline in their daily functioning (6, 52). Our findings are consistent with previous findings of a lower quality of life in a cohort of 62 adult SMA patients, which concluded that emotional distress was the most important determinant to both physical and mental quality of life (53). Mei Yao et al. also found a high prevalence of anxiety and depression in school-age SMA patients in the Chinese population (54). The current study was the first to report the high risk for anxiety and depression among Chinese adult patients with SMA and correlation between mental health and executive function. Hence, it is important to monitor the presence of depression or anxiety in adult patients with SMA and to consider psychological interventions to reduce these feelings resulting from impaired cognitive abilities and emotional responses to disease progression.

The effects of physical activity on cognition have been widely studied and these generally show a positive correlation. Such a relationship seems to be mediated through enhanced angiogenesis, increased oxygen saturation and glucose delivery, improved cerebral blood flow, and increased neurotransmitter levels (55). Previous studies demonstrated that physical activity could enhance cognition by modifying white matter integrity and activating key regions of the brain responsible for cognitive processes (56). Cognitive functioning has been closely linked to physical restrictions in SMA patients. The restricted interaction with environment because of severe physical condition and poor communication may reduce opportunities for SMA patients to develop important cognitive, affective, and psychomotor interactions (13). A study focusing on the survival pattern and functional status of SMA children revealed that the cognition scores of the real-life performance in daily activities of participants with SMA types III and II were higher than those with type I (36). The results probably reflected the fact that patients with greater disabilities depend more on caregivers’ help. Lucas et al. identified executive functions as the only cognitive domain correlated with disease severity, raising the question as to whether executive functions are especially important for patients with SMA (16). Our results also showed that SMA patients with greater motor difficulties had lower performance in executive function. It is likely that Patients’ inability to explore their surroundings due to muscle weakness hinders their cognitive development. As shown in a recent study, SMA patients with greater motor difficulties had lower performance in attention, especially for male patients (11). The author hypothesized that the existence of compensational mechanisms for physical disability does not occur at a universal level. During the compensation, the resource reallocation involved has decremental effects on specific cognitive domains such as executive function. Nevertheless, the neuropsychological tests in our study alone are not sufficient to fully assess general executive function in its entirety; this remains speculative and needs further investigation in future studies.

The study has several limitations. Firstly, our findings were limited by the small sample size, which may restrict the generalizability of our results. At the same time, SMA is a rare disease, and an untreated cohort is relatively difficult to recruit, but our sample size was similar compared with the recently published studies on adult SMA patients (11, 16). The imbalance of the sexes differed significantly within our patient cohorts; most of our patients were male and the results might not be representative of female patients. In SMA, past studies have suggested the presence of sex-specific modifiers on intrinsic brain pathology and cognitive adaptation mechanisms following physical dysfunction (57). Additionally, the present study serves as an exploratory investigation of associated factors. In order to comprehensively identify potential cognitive correlates, we refrained from adjusting for multiple comparisons in correlation analyses, which may potentially inflate the observed correlation between certain factors and executive function. A further limitation of this study was that the tests we used did not account for motor impairment, which may have influenced the results. It is reasonable that the degree of executive impairment varied among different investigations due to diverging degrees of action requirements and criteria. The ANT, for example, was not adjusted for patients with upper limb dysfunction, whose scores might be lowered consequently. Although all the participants demonstrated fluent finger movement and writing skills, considering fatigue is a core clinical feature of SMA, they may have experienced loss of dexterity in their fingers during the examination. Our findings suggested that executive function could also be associated with anxiety and depression, suggesting a more complex interaction of several factors. Thus, we could not rule out the possibility that the lower performance found in some executive tests might be due to physical disability or emotional impairment. Furthermore, the normative information adjusted by age and education on neuropsychological measures for the Chinese population is still scarce. The findings should be validated in a more extensive population. Finally, this was a cross-sectional design with data collected at a single time point. Nevertheless, we assumed that our cohorts were still representative of a subset of untreated adult SMA patients in China. The findings await validation by future longitudinal and population-based investigations. Moreover, although we cannot draw causal assumptions from cross-sectional data, the correlational findings help identify potential causal models.

## 5. Conclusion

In conclusion, our results indicated that the executive function in SMA patients seems to be selectively impaired. We also provide initial evidence that the executive function was associated with lower motor performance as well as anxiety and depression. These preliminary findings expand our perspective on the understanding of the disease and may have implications for the management of SMA patients in clinical practice. It should be noted that this impairment was mild and its clinical significance remains uncertain. Besides, physical disability should be taken into consideration in the design of neuropsychological evaluations. A better knowledge of brain involvement would improve the interpretation of clinical phenotypes and the personalization of rehabilitation programs supporting patients’ autonomies and quality of life. Further research is encouraged to verify our conclusion in a multicenter-based design with a large sample size and accounting for physical and emotional impairment.

## Data availability statement

The raw data supporting the conclusions of this article will be made available by the authors, without undue reservation.

## Ethics statement

The studies involving human participants were reviewed and approved by Anhui Medical University Ethics Committee. Written informed consent to participate in this study was provided by the participants.

## Author contributions

YH compiled background information and wrote the manuscript. LW, AL, and TL acquired and analyzed the data. YH, YJ, and CX were treating physicians. CX and KW revised the manuscript. All authors contributed to the article and approved the submitted version.
