# Supplementary material for: Cognitive impairment in Chinese adult patients with type III spinal muscular atrophy without disease-modifying treatment
Source: Front Neurol. 2023 Nov 3;14:1226043. doi: 10.3389/fneur.2023.1226043 (PMC10655145; doi:10.3389/fneur.2023.1226043)
Supplement: Supplementary file 1 [file Table_1.docx]

Supplementary Material

Cognitive impairment in Chinese adult patients with type III spinal muscular atrophy without disease-modifying treatment

Ying Hu ^1,2,3^, Ling Wei^1,2,3^, Aonan Li ^1,2,3^, Tingting Liu^1,2,3^, Yubao Jiang ^1,2,3^, Chengjuan Xie^1,2,3^, Kai Wang^1,2,3, *^

^1^ Department of Neurology, The First Affiliated Hospital of Anhui Medical University, Hefei, China,

^2^ The School of Mental Health and Psychological Sciences, Anhui Medical University, Hefei, China

^3^ Anhui Province Key Laboratory of Cognition and Neuropsychiatric Disorders, Hefei, China

*** Correspondence:**Corresponding Author
wangkai1964@126.com

# Supplementary Data

Supplementary Material should be uploaded separately on submission. Please include any supplementary data, figures and/or tables.

Supplementary material is not typeset so please ensure that all information is clearly presented, the appropriate caption is included in the file and not in the manuscript, and that the style conforms to the rest of the article.

# Supplementary Figures and Tables

For more information on Supplementary Material and for details on the different file types accepted, please see [here](https://www.frontiersin.org/guidelines/author-guidelines#supplementary-material).

**
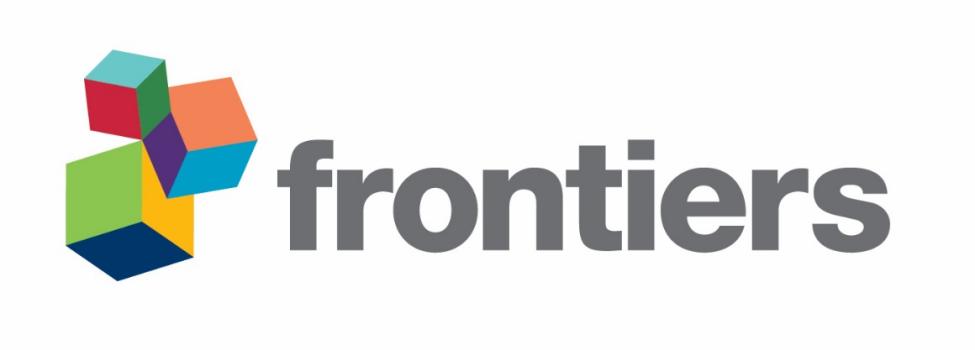
**

Supplementary Table **1** Correlation between clinical variables and neuropsychological cognitive tests in patients with SMA. The rho scores of Spearman correlations were listed in the table.

Abbreviations: MoCA, the Montreal Cognitive Assessment; SMN2, survival motor neuron 2 gene; HFMSE, Hammersmith Functional Motor Scale Expanded; RULM, Revised Upper Limb Module; 6MWT, 6 min walking test; AVLT, Auditory Verbal Learning Task; DS, Digital Span; VFT, Verbal Fluency Test; HAMD, Hamilton Depression Scale; HAMA, Hamilton Anxiety Scale; WCST, Wisconsin Card Sorting Task; RT, reaction time. Significance levels: ^*^ *p* ＜ 0.05; ^**^ *p* ＜ 0.01.

|  | Age | Disease duration | Age at onset | Education | MoCA | SMN2 opy number | HMFS | 6MWT | RULM | AVLT immediate | AVLT delayed | AVLT recognition | DS forward | DS backward | stroop interference | VF animals | VF fruits and vegetables | VF water | HAMD | HAMA | Alerting | Orienting | Executive | Mean RT | Accuracy | Categories Completed | Number of Trials | Correct Responses | Total Errors | Trials to 1st category | Perseverative Responses | Perseverative Errors | Non-perseverative Errors |
| --- | --- | --- | --- | --- | --- | --- | --- | --- | --- | --- | --- | --- | --- | --- | --- | --- | --- | --- | --- | --- | --- | --- | --- | --- | --- | --- | --- | --- | --- | --- | --- | --- | --- |
| Age | 1 |  |  |  |  |  |  |  |  |  |  |  |  |  |  |  |  |  |  |  |  |  |  |  |  |  |  |  |  |  |  |  |  |
| Disease duration | .518** | 1 |  |  |  |  |  |  |  |  |  |  |  |  |  |  |  |  |  |  |  |  |  |  |  |  |  |  |  |  |  |  |  |
| Age at onset | .459* | -0.204 | 1 |  |  |  |  |  |  |  |  |  |  |  |  |  |  |  |  |  |  |  |  |  |  |  |  |  |  |  |  |  |  |
| Education | 0.028 | -.480* | .417* | 1 |  |  |  |  |  |  |  |  |  |  |  |  |  |  |  |  |  |  |  |  |  |  |  |  |  |  |  |  |  |
| MoCA | -0.353 | -0.042 | 0.136 | 0.127 | 1 |  |  |  |  |  |  |  |  |  |  |  |  |  |  |  |  |  |  |  |  |  |  |  |  |  |  |  |  |
| SMN2 copy number | 0.321 | -0.098 | .540* | .671** | -0.082 | 1 |  |  |  |  |  |  |  |  |  |  |  |  |  |  |  |  |  |  |  |  |  |  |  |  |  |  |  |
| HMFS | 0.141 | -0.07 | .835** | .497* | 0.318 | 0.299 | 1 |  |  |  |  |  |  |  |  |  |  |  |  |  |  |  |  |  |  |  |  |  |  |  |  |  |  |
| 6MWT | -.546* | -0.061 | -0.17 | .577* | 0.503 | 0.279 | .892** | 1 |  |  |  |  |  |  |  |  |  |  |  |  |  |  |  |  |  |  |  |  |  |  |  |  |  |
| RULM | .511* | 0.021 | .952** | .392* | 0.014 | 0.392 | .684** | 0.086 | 1 |  |  |  |  |  |  |  |  |  |  |  |  |  |  |  |  |  |  |  |  |  |  |  |  |
| AVLT immediate | -.441* | -0.144 | 0.038 | 0.242 | 0.332 | 0.236 | 0.032 | 0.182 | 0.106 | 1 |  |  |  |  |  |  |  |  |  |  |  |  |  |  |  |  |  |  |  |  |  |  |  |
| AVLT delayed | -0.176 | -0.198 | 0.234 | .414* | 0.08 | 0.303 | 0.103 | -0.034 | 0.247 | .870** | 1 |  |  |  |  |  |  |  |  |  |  |  |  |  |  |  |  |  |  |  |  |  |  |
| AVLT recognition | -0.325 | -.411* | 0.035 | .493* | -0.007 | .497* | 0.014 | 0.28 | 0.111 | .582** | .472* | 1 |  |  |  |  |  |  |  |  |  |  |  |  |  |  |  |  |  |  |  |  |  |
| DS forward | -0.216 | -0.137 | -0.063 | 0.228 | 0.262 | 0.018 | 0.102 | -0.021 | 0.008 | .471* | .609** | 0.089 | 1 |  |  |  |  |  |  |  |  |  |  |  |  |  |  |  |  |  |  |  |  |
| DS backward | -0.348 | -0.184 | -0.117 | .476* | 0.327 | 0.114 | 0.21 | 0.101 | -0.119 | .573** | .651** | 0.29 | .854** | 1 |  |  |  |  |  |  |  |  |  |  |  |  |  |  |  |  |  |  |  |
| stroop interference | 0.207 | 0.009 | -0.055 | -0.033 | -0.338 | 0.410 | -0.308 | 0.291 | -0.211 | -0.273 | -0.35 | 0.197 | -.627** | -.514** | 1 |  |  |  |  |  |  |  |  |  |  |  |  |  |  |  |  |  |  |
| VF animals | -0.202 | 0.102 | -0.142 | 0.087 | -0.009 | 0.097 | -0.162 | -0.391 | -0.01 | .740** | .806** | 0.341 | .448* | .600** | -0.321 | 1 |  |  |  |  |  |  |  |  |  |  |  |  |  |  |  |  |  |
| VF fruits and vegetables | 0.12 | 0.153 | -0.202 | -0.532 | 0.241 | -0.469 | -0.319 | -0.428 | -0.393 | -0.06 | -0.075 | -.654** | 0.033 | 0.07 | -0.257 | 0.2 | 1 |  |  |  |  |  |  |  |  |  |  |  |  |  |  |  |  |
| VF water | 0.091 | -0.177 | 0.281 | 0.334 | -0.059 | 0.302 | 0.008 | -0.636 | 0.099 | .420* | .647** | 0.107 | .410* | .554** | -0.152 | .621** | 0.289 | 1 |  |  |  |  |  |  |  |  |  |  |  |  |  |  |  |
| HAMD | 0.183 | 0.263 | -0.163 | -.423* | -.548** | 0.226 | -0.227 | -0.253 | -0.068 | -0.17 | -0.109 | -0.245 | -0.373 | -.438* | .475* | 0.069 | -0.009 | -0.044 | 1 |  |  |  |  |  |  |  |  |  |  |  |  |  |  |
| HAMA | 0.33 | 0.153 | 0.16 | -0.103 | -.422* | .0.552 | -0.087 | -0.241 | 0.224 | 0.027 | 0.135 | 0.063 | -0.218 | -0.273 | .560** | 0.183 | -0.24 | 0.164 | .828** | 1 |  |  |  |  |  |  |  |  |  |  |  |  |  |
| Alerting | 0.072 | -0.181 | 0.253 | 0.257 | .431* | 0.338 | -0.021 | .578* | 0.14 | 0.228 | 0.087 | 0.375 | 0.042 | -0.028 | 0.228 | -0.197 | -0.232 | -0.149 | -.487* | -0.135 | 1 |  |  |  |  |  |  |  |  |  |  |  |  |
| Orienting | -0.014 | 0.044 | -0.186 | -0.297 | -0.109 | 0.18 | -0.109 | 0.22 | -0.008 | -0.134 | -0.259 | 0.027 | 0.177 | -0.115 | 0.167 | -.421* | -0.381 | -0.344 | 0.144 | 0.156 | 0.062 | 1 |  |  |  |  |  |  |  |  |  |  |  |
| Executive | -0.106 | 0.268 | -0.27 | -.474* | -0.174 | -0.085 | -0.21 | -0.321 | -0.321 | -0.304 | -0.308 | -0.298 | -0.216 | -0.256 | 0.287 | -0.062 | 0.08 | -0.259 | .692** | .553** | -.530** | 0.165 | 1 |  |  |  |  |  |  |  |  |  |  |
| Mean RT | 0.145 | 0.326 | 0.011 | -.614** | -0.207 | -0.053 | -0.278 | -0.002 | -0.01 | -0.153 | -0.259 | -0.36 | -.572** | -.732** | 0.318 | -0.215 | 0.189 | -0.322 | .715** | .454* | -0.202 | 0.12 | .436* | 1 |  |  |  |  |  |  |  |  |  |
| Accuracy | -0.167 | -.458* | 0.224 | 0.187 | 0.262 | -0.162 | 0.322 | -0.005 | 0.263 | -0.263 | -0.361 | 0.1 | -0.126 | -0.071 | -0.195 | -0.364 | -0.295 | -0.141 | -.487* | -.403* | 0.132 | 0.034 | -0.314 | -0.395 | 1 |  |  |  |  |  |  |  |  |
| Categories Completed | -0.225 | -0.357 | -0.074 | 0.241 | 0.12 | -0.056 | -0.207 | -0.328 | -0.178 | 0.328 | 0.382 | 0.19 | .457* | .549** | -0.131 | 0.376 | 0.139 | .627** | -.457* | -0.248 | 0.217 | -0.168 | -.435* | -.669** | 0.198 | 1 |  |  |  |  |  |  |  |
| Number of Trials | -0.085 | 0.309 | -0.031 | -0.03 | 0.091 | 0.29 | 0.323 | -.373 | 0.171 | 0.122 | -0.141 | 0.25 | -0.361 | -0.241 | 0.234 | -0.127 | -0.311 | -.495* | 0.327 | 0.264 | -0.101 | 0.279 | 0.325 | .426* | -0.134 | -.730** | 1 |  |  |  |  |  |  |
| Correct Responses | -0.124 | -0.081 | 0.378 | .431* | 0.366 | 0.371 | 0.215 | .607* | 0.28 | .598** | .402* | .577** | -0.196 | 0.11 | 0.213 | 0.296 | -0.098 | 0.261 | -0.275 | 0.027 | .418* | -0.335 | -0.302 | -0.108 | 0.055 | 0.23 | 0.271 | 1 |  |  |  |  |  |
| Total Errors | -0.051 | 0.322 | -0.289 | -0.325 | -0.15 | -0.005 | 0.185 | .536 | -0.006 | -0.236 | -0.37 | -0.128 | -0.26 | -0.341 | 0.093 | -0.294 | -0.226 | -.673** | .516** | 0.233 | -0.355 | .419* | .512** | .539** | -0.209 | -.872** | .787** | -0.366 | 1 |  |  |  |  |
| Trials to 1st category | 0.116 | .528** | -.538** | -.448* | -0.043 | -0.217 | -.435* | 0.204 | -0.586 | -0.078 | -0.184 | -0.315 | -0.187 | -0.173 | 0.087 | 0.027 | .592** | -0.179 | 0.214 | -0.108 | -0.004 | -0.143 | -0.007 | .420* | -.555** | -0.287 | 0.085 | -0.22 | 0.269 | 1 |  |  |  |
| Perseverative Responses | 0.246 | 0.15 | -0.022 | 0.065 | -0.166 | 0.257 | 0.269 | .523 | -0.004 | -.523** | -.580** | -0.069 | -0.329 | -0.349 | 0.392 | -.720** | -0.337 | -.564** | 0.173 | 0.062 | -0.03 | .535** | 0.209 | 0.217 | -0.016 | -.639** | .575** | -0.25 | .673** | 0.132 | 1 |  |  |
| Perseverative Errors | -0.031 | 0.287 | -0.222 | -0.247 | -0.06 | 0.007 | 0.282 | .393 | 0.094 | -0.328 | -.472* | -0.056 | -0.28 | -0.354 | 0.111 | -0.377 | -0.349 | -.776** | 0.363 | 0.182 | -0.24 | .421* | .534** | 0.376 | -0.001 | -.839** | .774** | -0.32 | .942** | 0.077 | .681** | 1 |  |
| Non-perseverative Errors | -0.11 | 0.389 | -.441* | -.391* | -0.158 | -0.218 | -0.181 | 0.216 | -0.296 | -0.14 | -0.285 | -0.206 | -0.291 | -0.293 | -0.075 | -0.04 | 0.179 | -.400* | .487* | 0.074 | -.523** | 0.137 | .409* | .616** | -0.246 | -.753** | .594** | -0.348 | .818** | .551** | 0.377 | .672** | 1 |
